# One Dose of *Staphylococcus aureus* 4C-Staph Vaccine Formulated with a Novel TLR7-Dependent Adjuvant Rapidly Protects Mice through Antibodies, Effector CD4^+^ T Cells, and IL-17A

**DOI:** 10.1371/journal.pone.0147767

**Published:** 2016-01-26

**Authors:** Francesca Mancini, Elisabetta Monaci, Giuseppe Lofano, Antonina Torre, Marta Bacconi, Simona Tavarini, Chiara Sammicheli, Letizia Arcidiacono, Bruno Galletti, Donatello Laera, Michele Pallaoro, Giovanna Tuscano, Maria Rita Fontana, Giuliano Bensi, Guido Grandi, Silvia Rossi-Paccani, Sandra Nuti, Rino Rappuoli, Ennio De Gregorio, Fabio Bagnoli, Elisabetta Soldaini, Sylvie Bertholet

**Affiliations:** 1 Novartis Vaccines and Diagnostics, S.r.l., Research Center, Siena, Italy; 2 Department of Biomedical Sciences, University of Padua, Padua, Italy; 3 Department of Biology and Biotechnologies “Charles Darwin”, University of Rome “La Sapienza”, Rome, Italy; 4 Department of Biotechnology, Chemistry and Pharmacy, University of Siena, Siena, Italy; Imperial College London, UNITED KINGDOM

## Abstract

A rapidly acting, single dose vaccine against *Staphylococcus aureus* would be highly beneficial for patients scheduled for major surgeries or in intensive care units. Here we show that one immunization with a multicomponent *S*. *aureus* candidate vaccine, 4C-Staph, formulated with a novel TLR7-dependent adjuvant, T7-alum, readily protected mice from death and from bacterial dissemination, both in kidney abscess and peritonitis models, outperforming alum-formulated vaccine. This increased efficacy was paralleled by higher vaccine-specific and α-hemolysin-neutralizing antibody titers and Th1/Th17 cell responses. Antibodies played a crucial protective role, as shown by the lack of protection of 4C-Staph/T7-alum vaccine in B-cell-deficient mice and by serum transfer experiments. Depletion of effector CD4^+^ T cells not only reduced survival but also increased *S*. *aureus* load in kidneys of mice immunized with 4C-Staph/T7-alum. The role of IL-17A in the control of bacterial dissemination in 4C-Staph/T7-alum vaccinated mice was indicated by *in vivo* neutralization experiments. We conclude that single dose 4C-Staph/T7-alum vaccine promptly and efficiently protected mice against *S*. *aureus* through the combined actions of antibodies, CD4^+^ effector T cells, and IL-17A. These data suggest that inclusion of an adjuvant that induces not only fast antibody responses but also IL-17-producing cell-mediated effector responses could efficaciously protect patients scheduled for major surgeries or in intensive care units.

## Introduction

*Staphylococcus aureus* infections of the bloodstream or deep wound are a serious complication of major surgeries, including cardiothoracic and orthopedic surgery, resulting in significant morbidity and mortality [1, 2]. *S*. *aureus* is also the most commonly isolated microorganism from patients in intensive care units, which have mortality rates that reach 60% [3]. Due to methicillin-resistant *S*. *aureus* infections, up to one third of patients diagnosed with *S*. *aureus* bacteremia succumb even when treated with appropriate antibiotic therapy [4]. Therefore, a vaccine that provides rapid protection against *S*. *aureus* during the post-operative or intensive care period would address an important unmet medical need.

We recently developed a four-component *S*. *aureus* vaccine (4C-Staph) consisting of Hla_H35L_, EsxAB, FhuD2, and Csa1A recombinant proteins [5]. Hla_H35L_ is a genetically detoxified mutant of α-hemolysin (Hla) [6], a highly conserved exotoxin that plays a prominent role in early stages of invasive infections disrupting epithelial and endothelial barriers, contributing to pathogen-associated mortality [6, 7]. Immunization with Hla_H35L_ partially protected mice against staphylococcal pneumonia, peritonitis, and skin infections inducing functional antibodies neutralizing the lytic activity of native Hla [8–10]. Remarkably, however, Hla neutralization was not sufficient to eradicate *S*. *aureus* infection, suggesting that additional antigens are required for an efficacious vaccine [9]. EsxAB is a fusion of the two ESAT-6-like secreted virulence factors EsxA and EsxB associated to abscess formation, which may facilitate persistence and spread of the pathogen in the infected host [11–13]. FhuD2 is a lipoprotein involved in iron uptake and in early stages of invasive *S*. *aureus* infection [14–16], while Csa1A is a putative lipoprotein whose role in pathogenesis is under investigation [17]. We have recently shown that two doses of 4C-Staph vaccine formulated with alum protected against a panel of epidemiologically relevant *S*. *aureus* strains in kidney abscess, peritonitis, skin, and pneumonia mouse models of *S*. *aureus* infection [5].

Adjuvants enhance and accelerate adaptive immune responses toward a co-administered antigen, while also directing the quality of the immune response [18, 19]. Three major types of cell-mediated effector immunity meant to optimally respond to distinct threats have been identified: type 1 protects against intracellular pathogens and comprises IFN-γ-producing cells (e.g. Th1 cells), type 2 protects against helminths and comprises IL-4/IL-13-producing cells (e.g. Th2), while type 3 protects against extracellular bacteria and fungi and comprises IL-17-producing cells (e.g. Th17) [20]. Aluminum salts-based adjuvants, which are included in many licensed vaccines, preferentially induce type 2 responses while agonists of Toll-like receptors (TLRs), a family of receptors that recognize pathogen-associated molecular patterns [21, 22], induce mainly type 1 responses [18]. The potential of small molecule immune potentiators (SMIPs) targeting TLR7, an endosomal TLR that recognizes single-stranded RNAs, as vaccine adjuvants has been shown in pre-clinical settings, especially for Imiquimod. However, systemic activation induced by these SMIPs has posed safety issues [23]. The rational design of SMIP.7-10, a novel TLR7 agonist that can be stably adsorbed to alum (T7-alum), minimized systemic exposure and inflammation while retained *in vivo* potency [24].

Therefore, in this study, we investigated whether single immunization with 4C-Staph/T7-alum conferred prompt protection against *S*. *aureus* infection. We found that this was indeed the case and we elucidated the immune mechanisms involved.

## Materials and Methods

### Vaccine formulations and animal immunizations

Vaccine antigens, Hla_H35L_, EsxAB, FhuD2, and Csa1A, were amplified by PCR from *S*. *aureus* NCTC8325 strain and cloned as tagless constructs. Antigens were purified and adsorbed to alum by incubation with aluminum hydroxide (alum) with or without SMIP-7.10 (T7), at pH 6.5–7.0 and osmolality 0.308±0.060 Osm/Kg, with slow stirring for a few hours at room temperature (RT) [5]. One dose of vaccine (100 μl) consisted in 10 μg of each antigen adsorbed to 2 mg/ml alum alone (4C-Staph/alum) or together with 50 μg SMIP-7.10 (4C-Staph/T7-alum). Formulations were adjusted to physiological ranges of pH and isotonicity and had an endotoxin content ˂1.5 European U/ml.

Five-week old female BALB/c (Charles River Laboratories) or J_H_ (Taconic) mice were used. For active immunization, mice were immunized i.m. (50 μl/hind leg quadriceps). Control mice received equal amounts of alum, or T7-alum. For passive immunization, pools of sera were prepared from BALB/c mice immunized once with 4C-Staph/T7-alum (immune serum) or T7-alum (control serum) by 32 days. Five-week-old naïve BALB/c mice were injected with 150 μl of immune or control serum into the tail vein the day before challenge with *S*. *aureus*.

### Bacterial inoculum and challenge

*S*. *aureus* Newman strain (kindly provided by Professor Schneewind, University of Chicago) was grown in tryptic soy broth (Difco Laboratories) at 37°C shaking at 250 rpm to an optical density at 600 nm (OD_600_) of 2. After centrifugation at 3,320 x g for 10 min., bacteria were washed with 50 ml of phosphate-buffered saline (PBS, pH 7.4; Invitrogen) and then diluted in PBS to obtain the required infectious dose/mouse. Inocula were verified experimentally by plating on tryptic soy agar and enumeration of the colony forming units (CFU) the day after.

Kidney abscess model: mice were challenged i.v. with 100 μl of a sublethal dose of bacteria (~ 2×10^7^ CFU). After 4 days, mice were euthanized; both kidneys were removed, homogenized in pool in 2 ml PBS, 2-fold serially diluted, and plated in duplicate for CFU counts.

Peritonitis model: mice were challenged i.p. with 100 μl of a LD_80_ of bacteria (~5 x 10^8^ CFU). Mice were monitored daily for 15 or 30 days, as indicated. Mice that survived were euthanized and CFU in kidneys were enumerated as described above.

### Quantification of 4C-Staph-specific and Hla-neutralizing antibodies

Vaccine-specific antibody titers were measured by Luminex (Luminex^®^ 200 TM) at a fixed serum dilution. Hla_H35L_, FhuD2, EsxAB and Csa1A purified proteins were covalently conjugated to the free carboxyl groups of xMAP multi-analyte microspheres (Luminex Corporation). Antigen-specific antibodies were revealed using PE-labeled secondary antibodies. For total IgG, median fluorescence intensity (MFI) values were converted into relative Luminex units (RLU)/ml using a mouse serum as standard.

Hla neutralizing antibodies were quantified with a rabbit red blood cells (RBCs)-based functional assay. Serial 2-fold dilutions of pooled mouse sera (n = 8/pool) were incubated with 12.5 nM Hla (Sigma) in triplicates. After 30 min at 37°C in agitation (350 rpm), RBCs (Emozoo Snc.) were added and incubation prolonged for 30 min without shaking. Then plates were centrifuged for 5 min at 1,000 x g and the OD_540_ of supernatants was determined with a SpectraMax^®^ 340PC384 Absorbance Microplate Reader (Molecular Devices). Percent hemolysis was calculated using as denominator the OD_540_ obtained following lysis of rabbit RBCs without mouse serum. Hla neutralizing titers were expressed as the reciprocal of the serum dilution inhibiting 50% of Hla hemolysis (ED_50_) given by native Hla, calculated using a "log (agonist) vs. normalized response" nonlinear regression curve (GraphPad Software, Inc.).

### Intracellular cytokine staining

Splenocytes were isolated 12 days after vaccination, plated at 1-2x10^6^ cells/well in 96-well plates, and stimulated with vaccine proteins (Hla_H35L_, EsxAB, FhuD2 and Csa1A, 10 μg/ml each) together with anti-CD28 and anti-CD49d mAb (2 μg/ml each, BD Biosciences) at 37°C for 16–18 h. Brefeldin A (5 μg/ml) was added for the last 4 h. The cells were then stained with Live/DeadYellow (Invitrogen), fixed and permeabilized with Cytofix/Cytoperm (BD Biosciences), washed in Perm/Wash buffer (BD Biosciences), incubated with anti-CD16/CD32 Fc block (BD Biosciences) for 20 min at RT, and stained with the following fluorochrome-conjugated mAbs anti: CD3-PerCP Cy5.5, CD4-V500, IFN-γ-PE, IL-2-APC, TNF-AF700, CD44-V450 (BD Pharmingen), CD8-PE Texas Red (Invitrogen), IL-17A-PE Cy7, IL-4-AF488 and IL-13-AF488 (eBioscience) in Perm/Wash buffer (BD Biosciences) for 20 min at RT, washed twice in Perm/Wash buffer, suspended in PBS. Samples were acquired on a LSRII special order flow cytometer (BD Biosciences) and T-cell responses were analyzed using FlowJo software (TreeStar) applying the gating strategy described in S1 Fig.

### Depletion of effector CD4^+^ T cells

To deplete effector CD4^+^ T cells, mice were vaccinated on day 0, injected twice i.p. with 100 μg of a rat anti-CD4 mAb (clone GK1.5; Areta International) or isotype-matched control antibody (isot. ctr., rat IgG2b; R&D System) on day 6 and 8, and challenged with *S*. *aureus* on day 10. CD4^+^ cell depletion efficiency was evaluated by flow cytometry on heparinized blood taken the day before infection (day 9). For this purpose, RBCs were lysed with RBCs lysis buffer (Biolegend). White blood cells were stained with Live/Dead Yellow (Invitrogen), incubated with anti-CD16/CD32 Fc block (BD Biosciences), and stained with: anti-CD3-PerCP Cy5.5, (BD Pharmingen), anti-CD8-PE TexasRed (Invitrogen), and anti-CD4-Pacific Blue (Invitrogen) in PBS 0.1% BSA for 20 min at room temperature. Cells were fixed with Cytofix (BD Biosciences), suspended in PBS and analyzed by LSR II flow cytometer (BD Biosciences). No CD4^+^ T cells were detected in the peripheral blood indicating that depletion occurred efficiently (S2 Fig).

### *In vivo* cytokine neutralization

To neutralize IL-17A and/or IFN-γ *in vivo*, mice immunized with 4C-Staph/T7-alum or T7-alum were injected i.p. with 100 μg of a neutralizing mouse anti-IL-17A mAb (clone 17F3; BioXCell) and/or a neutralizing rat anti-IFN-γ mAb (clone XMG 1.2; BioXCell) every other day starting from 3 h prior to *S*. *aureus* challenge (day 12 after vaccination) until sacrifice. Control mice received the same amount of isot. ctr. antibodies (mouse IgG1 and/or rat IgG1; BioXCell).

### Statistical analysis

Log-rank test was used to evaluate differences in survival of infected mice. One-way ANOVA and Sidak’s multiple comparisons test were used to evaluate differences in the bacterial load in kidneys of infected mice and differences in CD4^+^ T-cell responses to vaccination when more than two groups were compared. Comparisons between frequencies of cytokine-producing CD4^+^ T cells in response to 4C-Staph/alum or 4C-Staph/T7-alum vaccination were performed using a Student's *t* test and a partial permutation test. For total IgG titers, Kruskall-Wallis and Dunn's multiple comparisons tests were used to evaluate differences in vaccine-specific antibody titers. Statistical analyses were performed using GraphPad Prism 6 software (GraphPad Software, Inc.) and SPICE version 5.1 (http://exon.niaid.nih.gov).

### Ethics statement

All animal studies were carried out in compliance with current Italian legislation on the care and use of animals in experimentation (Legislative Decree 26/2014, authorizations 185/2011-B and 249/2011-B approved by the Italian Ministry of Health) and with the Novartis Animal Welfare Policy and Standards (authorizations AWB 201105 and AWB 201106). After infection, mice were monitored daily using a dedicated clinical score system based on the following parameters: piloerection, hunched posture, decreased mobility, rolling/twisting movements and weight loss. Mice were euthanized by cervical dislocation when they exhibited either one of the following humane endpoints: a loss of 20% bodyweight and/or moderate to severe signs of rolling/twisting movements that were pre-established in agreement with Novartis Animal Welfare Policies. All animals were provided environmental enrichment and food and water were offered *ad libitum*.

## Results

### One dose of 4C-Staph/T7-alum vaccine confers rapid and efficacious protection against *S*. *aureus*

We tested the efficacy of one dose of adjuvanted 4C-Staph vaccines in the mouse kidney abscess and peritonitis models. For this purpose, BALB/c mice were vaccinated i.m. with 4C-Staph/T7-alum or 4C-Staph/alum vaccines. Control groups of mice received T7-alum or alum. Twelve days after immunization, mice were challenged with *S*. *aureus* Newman strain.

**Fig 1:**
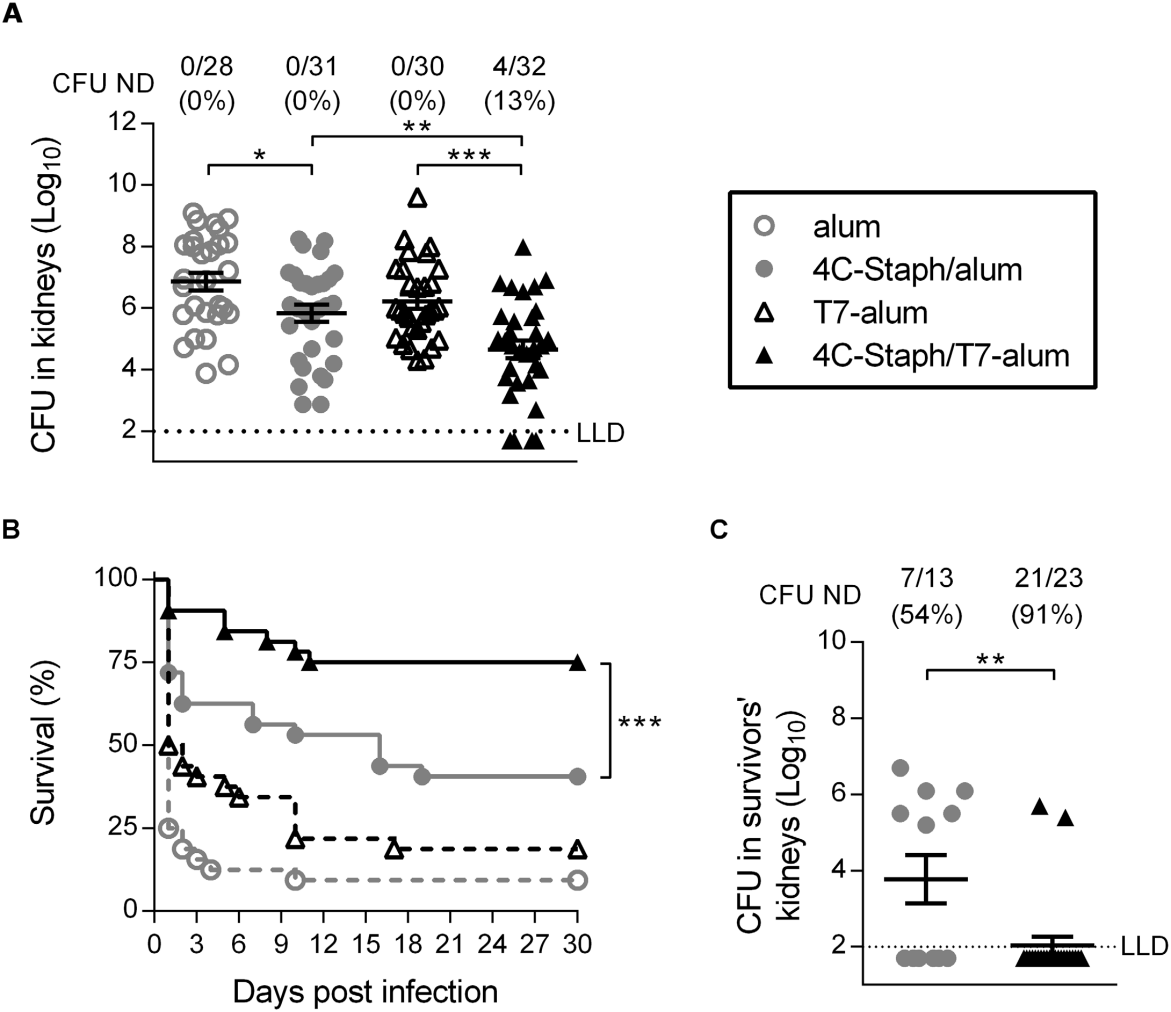
One dose of 4C-Staph/T7-alum vaccine protects better than 4C-Staph/alum in kidney abscess and peritonitis models of *S*. *aureus* infection. BALB/c mice were immunized once i.m. with 4C-Staph/T7-alum or 4C-Staph/alum. Control mice were injected with T7-alum or alum alone. After 12 days, mice were challenged with *S*. *aureus* Newman strain. (**A**) Kidney abscess model. Mice (n = 28–32) were injected i.v. with 2 x 10^7^ CFU. Four days later, both kidneys of each mouse were homogenized in pool and CFU enumerated. Each symbol represents one mouse, and data are the merge of three independent experiments. Mean ± SEM of each group are shown. The dotted line indicates the lower limit of detection (LLD). *p < 0.05, **p < 0.01 by one-way ANOVA and Sidak's multiple comparisons test. Number of survivors with non-detectable CFU (CFU ND) in kidneys/total number of survivors and corresponding percentages are reported above the graph. (**B-C**) Peritonitis model. Mice (n = 32) were injected i.p. with 5 x 10^8^ CFU. Survival was monitored for 30 days after challenge. Data are the merge of three independent experiments. ***p < 0.001 by Log-rank test. (**C**) Thirty days after *S*. *aureus* infection, survivors were euthanized, both kidneys were homogenized and CFU enumerated. Each symbol represents one mouse. Mean ± SEM of each group is shown. **p < 0.01 by unpaired Student *t* test, one-tailed. Number of survivors with CFU ND in kidneys/total number of survivors and corresponding percentages are reported above the graph.

In the kidney abscess model, mice were challenged i.v. with a sublethal dose of bacteria (2x10^7^ CFU). After 4 days mice were sacrificed and CFU in kidneys were enumerated. As shown in Fig 1A, 4C-Staph/T7-alum-vaccinated mice showed a 1.55 log_10_ reduction in CFU/kidneys compared to T7-alum-treated control mice (4.66 ± 0.28 and 6.21 ± 0.23 log_10_, mean ± SEM CFU/kidneys, respectively, *p* = 0.0003), and a 1.14 log_10_ reduction in CFU/kidneys compared to 4C-Staph/alum-immunized animals (5.80 ± 0.28 log_10_ CFU/kidneys, *p* = 0.0080). Remarkably, 4 out of 32 mice vaccinated with 4C-Staph/T7-alum had no detectable bacteria in kidneys compared to 0 out of 31 mice in the 4C-Staph/alum group.

In the peritonitis model, which can be used to study the spread of bacteria into the bloodstream [9], mice were challenged i.p. (5x10^8^ CFU) with a lethal dose of *S*. *aureus* and their survival was monitored for 30 days. One month after infection, 75% of mice immunized with 4C-Staph/T7-alum survived *S*. *aureus* challenge compared to 41% of mice immunized with 4C-Staph/alum (*p* = 0.0058, Fig 1B). Furthermore, mice vaccinated with 4C-Staph/T7-alum that survived had significantly less bacteria in kidneys (1.7 log_10_ reduction, *p* = 0.0091) and 91% of them had no detectable bacteria in kidneys as compared to survivors in the 4C-Staph/alum group (54%, Fig 1C).

Taken together, these data showed that single dose of 4C-Staph/T7-alum vaccine considerably reduced bacterial load in kidneys upon *S*. *aureus* i.v. challenge and not only allowed the vast majority of mice to survive after i.p. challenge, but also promoted efficient control of bacteria in kidneys of survivors.

### 4C-Staph/T7-alum vaccine rapidly induces functional and protective antibodies

**Fig 2:**
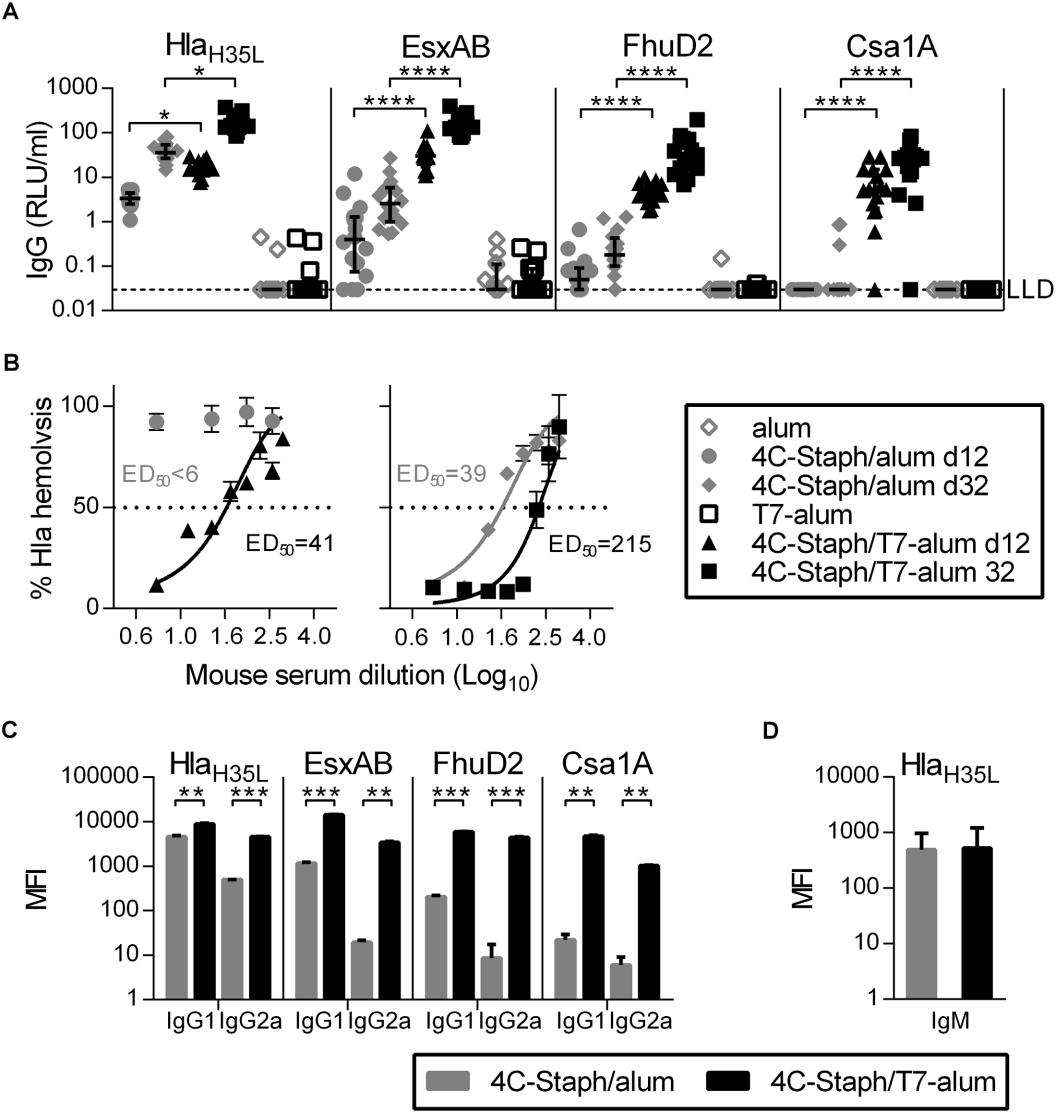
One dose of 4C-Staph/T7-alum induces functional antibodies. BALB/c mice (n = 16) were immunized once with 4C-Staph/T7-alum or 4C-Staph/alum. Control mice were injected with T7-alum or alum alone. (**A**) Vaccine-specific serum IgG titers measured 12 (d12) and 32 (d32) days after vaccination. IgG concentrations in control sera (open symbols) are reported only for d32. Each symbol represents one mouse, and data are the merge of two independent experiments. Median with interquartile range of each group is also shown. *p < 0.05, ****p < 0.0001 by Kruskal-Wallis test and Dunn's multiple comparisons test. (**B**) Hla neutralizing activity of pooled sera from vaccinated mice (n = 16, same animals as in A) was assessed on rabbit RBCs and expressed as effective dilution that neutralized 50% of Hla lytic activity (ED_50_). No hemolysis inhibition was detected (ED_50_ < 6) in pre-immune sera or in sera from adjuvant-treated mice. Lack of overlap in the 95% confidence intervals between the ED_50_ of sera from mice vaccinated with 4C-Staph/alum (30.3 to 51.4) vs. 4C-Staph/T7-alum (114.6 to 318.7) by 32 days indicates a difference significant with *p* < 0.05. Bars represent SEM. (**C**) Vaccine-specific IgG1 and IgG2a. Columns represent median MFI with interquartile range of pooled sera from vaccinated mice (n = 16, same pools as in B) bled at d32. **p < 0.01, ***p < 0.001 by unpaired Student *t* test, two-tailed. (**D**) Hla_H35L_-specific IgM. Columns represent median MFI with interquartile range of sera from vaccinated mice (n = 12) bled at d12. IgM specific for EsxAB, FhuD2 and Csa1A were at the limit of detection (data not shown). Data shown are the merge of two independent experiments.

The induction of antibodies specific for each component of the vaccine by 4C-Staph/T7-alum and 4C-Staph/alum was evaluated 12 and 32 days after vaccination. IgG specific for Hla_H35L_, EsxAB, FhuD2, and Csa1A were detected in sera from all mice vaccinated with 4C-Staph/T7-alum by day 12 and further increased by day 32, with the exception of one mouse that had no detectable anti-Csa1A IgG (Fig 2A). In contrast, only Hla_H35L_-specific IgG were detected in all sera from mice immunized with 4C-Staph/alum 12 days after vaccination, while EsxAB- and FhuD2-specific IgG were detected only in some sera, and Csa1A-specific IgG were not found. By day 32, Hla_H35L_- and EsxAB-specific IgG were found in sera from all 4C-Staph/alum vaccinated mice, while FhuD2- and Csa1A-specific IgG were found only in some sera. Higher levels of IgG1 and IgG2a specific for each vaccine component were found in sera of mice vaccinated with 4C-Staph/T7-alum as compared to sera of mice vaccinated with 4C-Staph/alum (Fig 2C), while no differences in vaccine-specific IgM levels were observed (Fig 2D). In addition, Hla neutralizing titers were measurable in sera from mice vaccinated with 4C-Staph/T7-alum by day 12 (ED_50_ = 41) and increased by 5-fold at day 32 (ED_50_ = 215), while Hla-neutralizing activity was detected in sera from mice vaccinated with 4C-Staph/alum at d32 (ED_50_ = 39) but not at day 12 (ED_50_ < 6, Fig 2B). Altogether, these data show that single dose of 4C-Staph/T7-alum vaccine readily induced not only seroconversion of vaccinated mice against each 4C-Staph component but also Hla-neutralizing antibodies.

**Fig 3:**
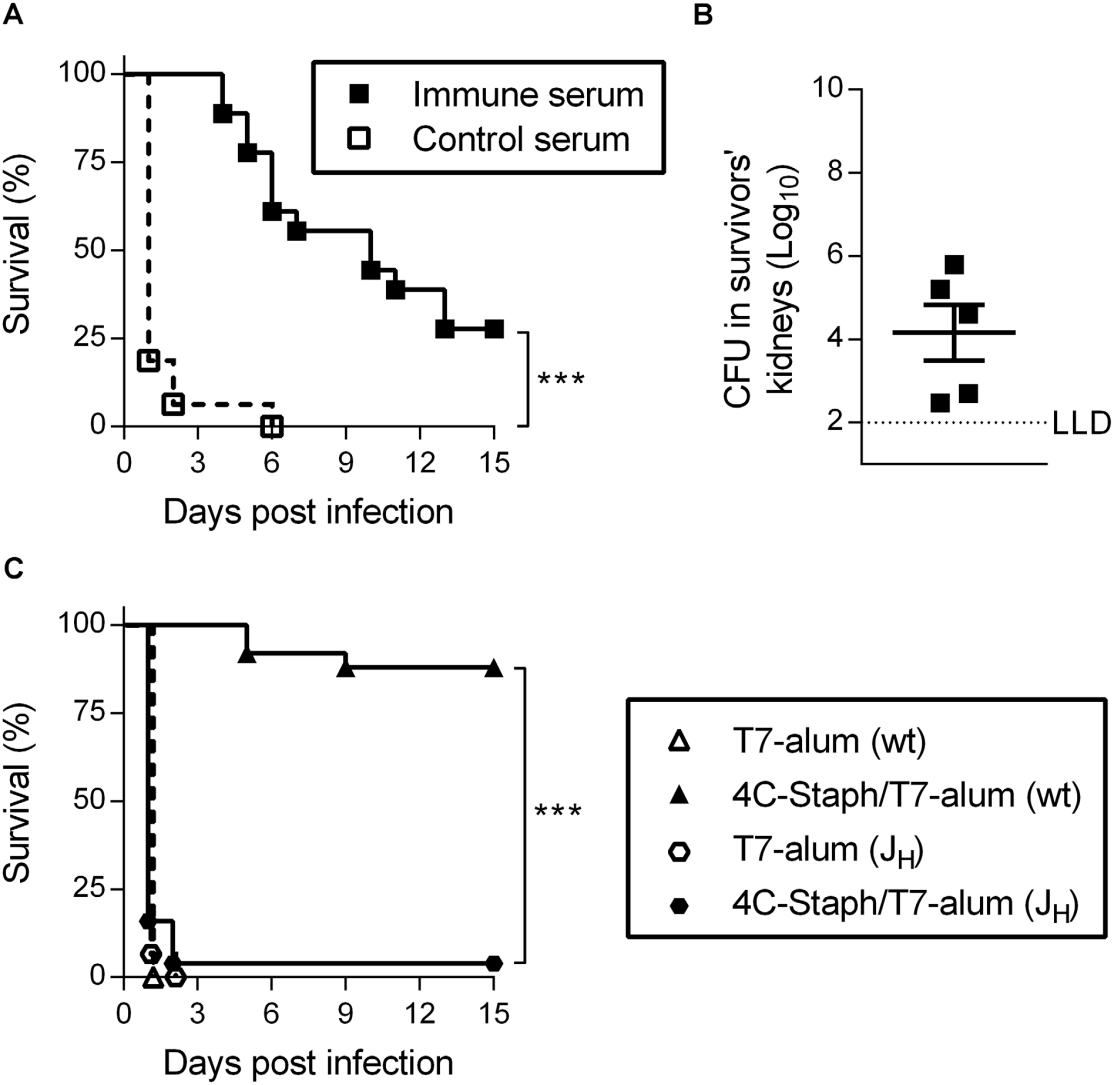
One dose of 4C-Staph/T7-alum induces protective antibodies. Sera from mice immunized with 4C-Staph/T7-alum (immune serum), or T7-alum as negative control (control serum), by 32 days were pooled and injected i.v. (150 μl/mouse) in naïve BALB/c mice (n = 16) 24 h before i.p. challenge with *S*. *aureus*. **(A**) Survival was monitored for 15 days post challenge. Data are the merge of two independent experiments. ***p < 0.001 by Log-rank test. **(B**) Fifteen days after *S*. *aureus* infection, survivors were euthanized, both kidneys were homogenized and CFU enumerated. Each symbol represents one mouse. **(C**) B cell/antibody-deficient J_H_ mice, or BALB/c (wt) as control, were immunized with 4C-Staph/T7-alum or T7-alum alone. Twelve days after vaccination, mice (n = 25 for 4C-Staph/T7-alum; n = 15 for T7-alum) were challenged i.p. with *S*. *aureus* and their survival was monitored for 15 days. Data are the merge of four independent experiments. ***p < 0.001 by Log-rank test.

To determine if antibodies induced by 4C-Staph/T7-alum vaccination were protective against a lethal challenge with *S*. *aureus*, sera collected from mice 32 days after immunization with 4C-Staph/T7-alum (or T7-alum, as control) were passively transferred into naïve mice one day before i.p. infection. All mice that received immune serum were protected from death during the first 3 days after infection (Fig 3A). Thereafter, the survival gradually declined to become 28% at day 15, when all survivors had bacteria in kidneys (Fig 3B). In contrast, 100% of mice that received control sera died by day 6 after infection. Finally, we showed that 4C-Staph/T7-alum vaccination required B cells to protect mice against *S*. *aureus* infection by using J_H_ mice, which lack mature B cells and antibodies [25]. Within two days after i.p. challenge, 96% of J_H_ mice vaccinated with 4C-Staph/T7-alum succumbed to the infection while 88% of BALB/c mice were still alive 15 days after infection (*p* < 0.0001, Fig 3C).

Overall these data suggest that antibodies induced by 4C-Staph/T7-alum vaccine are necessary and sufficient to protect against early death caused by *S*. *aureus* i.p. infection.

### 4C-Staph/T7-alum induces vaccine-specific Th1 and Th17 cells

**Fig 4:**
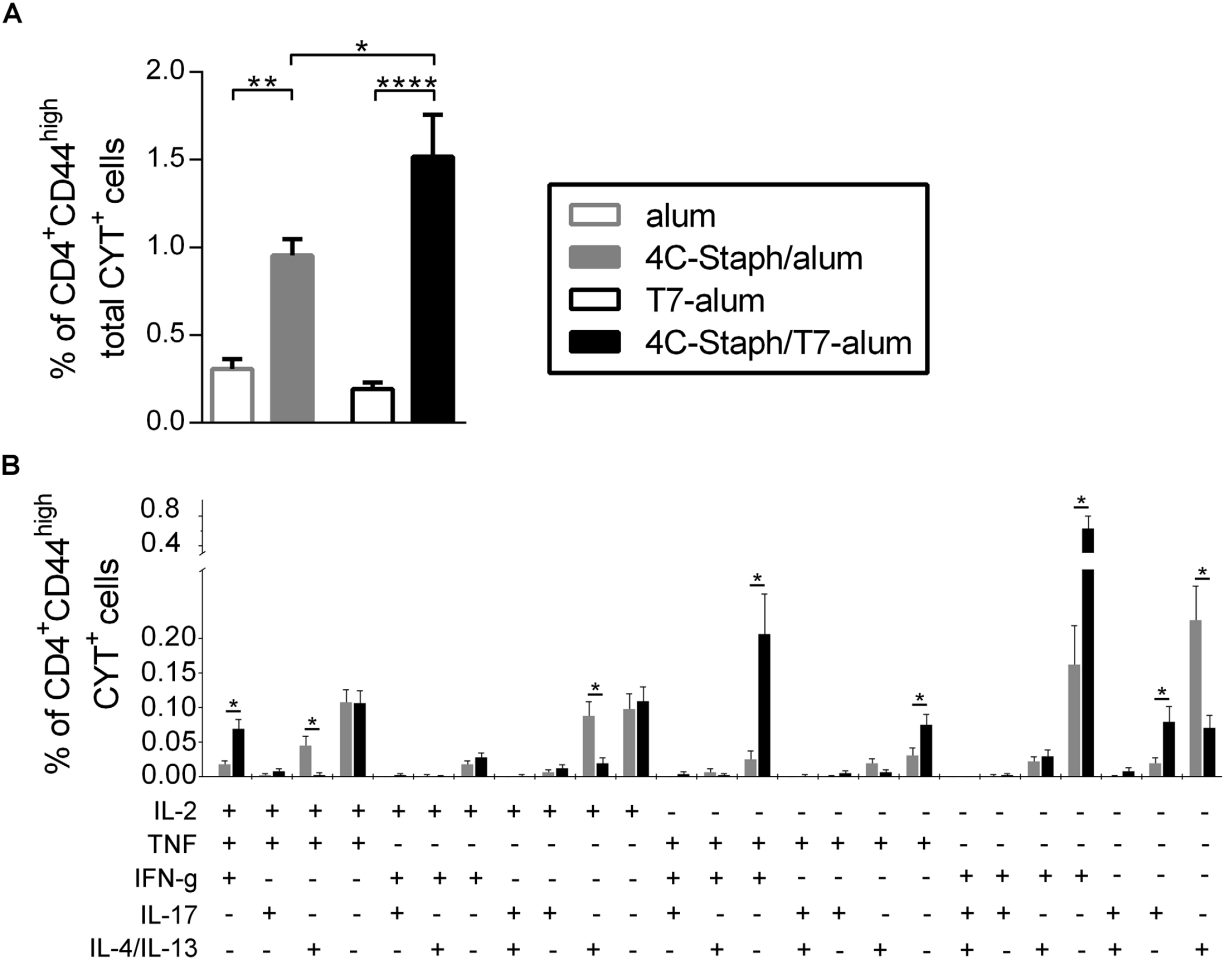
Magnitude and quality of vaccine-specific CD4^+^ T-cell responses induced by 4C-Staph/T7-alum and 4C-Staph/alum. Splenocytes from single mice (n = 16) vaccinated with 4C-Staph/T7-alum, 4C-Staph-alum, T7-alum or alum by 12 days were stimulated or not with vaccine antigens *in vitro*, stained and analyzed by intracellular cytokine staining. CD4^+^CD44^high^ T cells producing IL-2, TNF, IL-4/IL-13, IFN-γ or IL-17A were identified (see S1 Fig for gating strategy). The response of unstimulated cells was subtracted from that of stimulated cells. Data are the merge of four independent experiments. (**A**) Percentages of CD4^+^CD44^high^ T cells producing any combination of IL-2, TNF, IL-4/IL-13, IFN-γ or IL-17A in response to vaccine protein stimulation (CD4^+^CD44^high^ total CYT^+^ cells) were calculated applying Boolean gates. Bars represent mean ± SEM. *p < 0.05, **p < 0.01, ***p < 0.001 by one-way ANOVA and Sidak post-test. (**B**) Percentages of CD4^+^CD44^high^ T cells producing IL-2, TNF, IL-4/IL-13, IFN-γ and/or IL-17A in each of the possible combinations (CD4^+^CD44^high^ CYT^+^) in response to vaccine proteins stimulation calculated applying Boolean gates. No cells expressing more than 3 cytokines at once were detected. Bars represent mean ± SEM. *p < 0.05 by unpaired Student *t* test, two-tailed, and a partial permutation test.

Both human clinical observations and animal models support the concept that CD4^+^ T cells and Th17 cells in particular play a role in protection against *S*. *aureus* infection [26, 27]. Therefore, we evaluated the magnitude and the quality of vaccine-specific CD4^+^ T-cell responses induced by 4C-Staph/T7-alum and 4C-Staph/alum. For this purpose, splenocytes from mice immunized by 12 days were stimulated with 4C-Staph proteins *in vitro*. Vaccine-specific CD4^+^CD44^high^ T cells were identified and characterized at the single-cell level by polychromatic intracellular flow cytometry based on their ability to produce IL-2, TNF, IL-4/IL-13, IFN-γ and/or IL-17A. As shown in Fig 4A, the frequencies of CD4^+^CD44^high^ T cells producing any of these cytokines alone or in combination (total CYT^+^ cells) were higher in 4C-Staph/T7-alum than in 4C-Staph/alum vaccinated mice. Analyses of the quality of vaccine-specific CD4^+^CD44^high^CYT^+^ T cells revealed comparable frequencies of IL-2^+^ and IL-2^+^TNF^+^ cells amongst the two vaccines, while 4C-Staph/T7-alum induced higher percentages of TNF^+^ cells. In addition, 4C-Staph/T7-alum induced higher frequencies of Th1 cells (IFN-γ^+^ and TNF^+^IFN-γ^+^) and Th17 cells (IL-17A^+^), but lower percentages of Th2 cells (IL-4^+^/IL-13^+^, IL-2^+^IL-4^+^/IL-13^+^ and IL-2^+^TNF^+^IL-4^+^/IL-13^+^) than 4C-Staph/alum (Fig 4B). Interestingly, 4C-Staph/T7-alum also induced higher percentages of polyfunctional IL-2^+^TNF^+^IFN-γ^+^ CD4 T cells.

These results demonstrated that one dose of 4C-Staph/T7-alum induced higher frequencies of vaccine-specific cytokine-producing CD4^+^ T cells and polarized the CD4^+^ T cells more towards a Th1/Th17 phenotype as compared to 4C-Staph/alum.

### CD4^+^ T cells induced by 4C-Staph/T7-alum contribute to the protection against *S*. *aureus*

**Fig 5:**
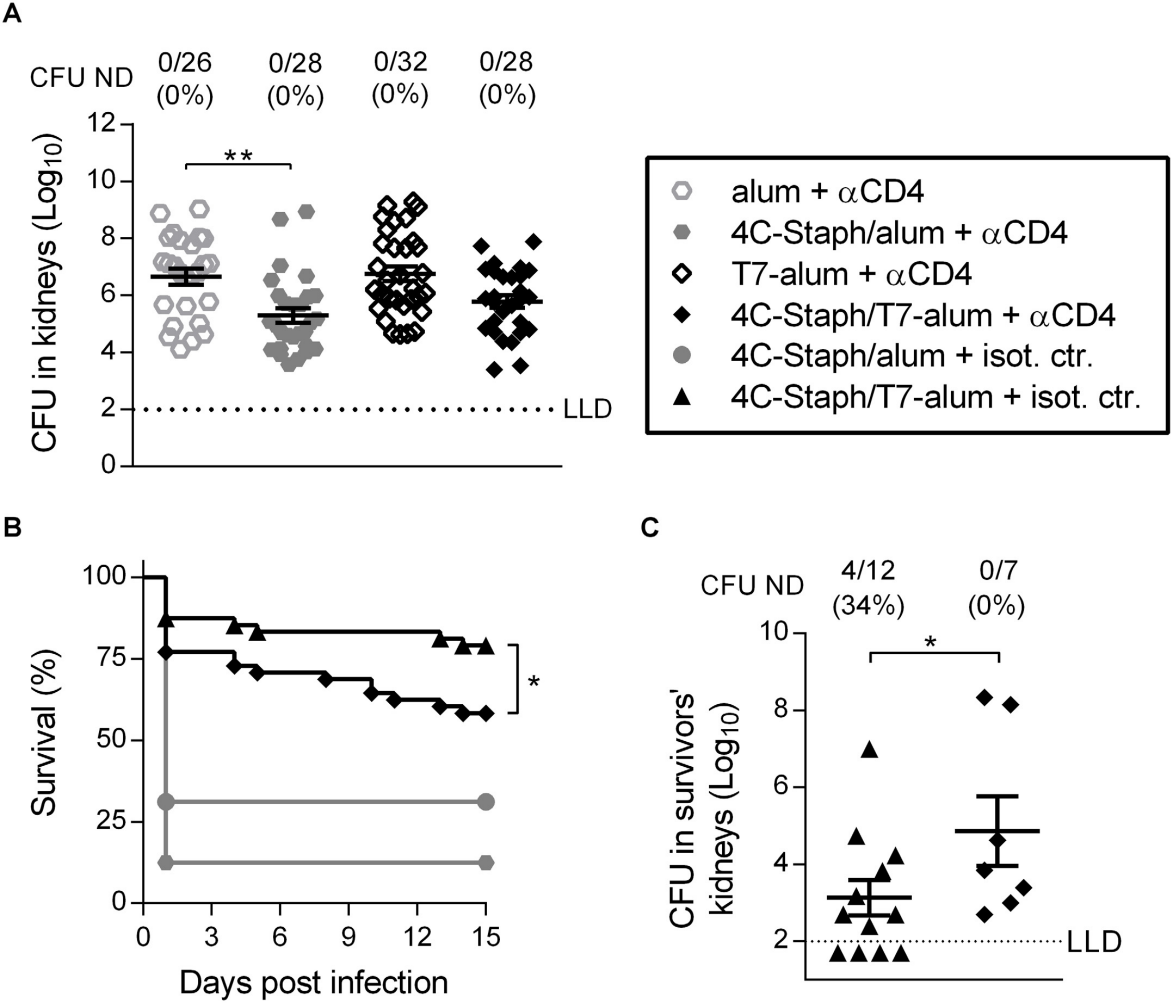
One dose of 4C-Staph/T7-alum vaccine induces protective CD4^+^ effector T cells. BALB/c mice injected once with 4C-Staph/alum, 4C-Staph/T7-alum, alum or T7-alum, were injected i.p. with an anti-CD4 (αCD4) or isotype control (isot. ctr.) on day 6 and 8 after vaccination. (**A**) Ten days after vaccination, mice (n = 26–32) were challenged i.v. with *S*. *aureus*. Four days later, the kidneys of each mouse were homogenized in pool and CFU enumerated. Each data point represents a mouse, data are the merge of three independent experiments. Mean ± SEM is reported. The dotted line indicates the lower limit of CFU detection. **p < 0.01 by one-way ANOVA and Sidak's multiple comparisons test. (**B-C**) Ten days after vaccination mice were challenged i.p. with *S*. *aureus*. (**B**) Survival was monitored for 15 days after challenge (n = 48). The difference between αCD4 and isot. ctr. was statistically significant (*p < 0.05) for mice vaccinated with 4C-Staph/T7-alum but not for mice vaccinated with 4C-Staph/alum by Log-rank test. (**C)** Fifteen days after *S*. *aureus* infection, survivors were euthanized, both kidneys homogenized and bacteria enumerated as CFU. Each data point represents a mouse. Data from a representative experiment out of 4 (n = 16) are shown as mean ± SEM. *p < 0.01 by Student *t* test, one-tailed unpaired. Number of survivors with CFU ND in kidneys/total number of survivors and corresponding percentages are reported above the graph.

To assess if vaccine-specific CD4^+^ T cells play a role in protection, mice were treated with an anti-CD4 mAb 6 and 8 days after immunization with 4C-Staph/alum or 4C-Staph/T7-alum to allow T cell-dependent antibody production, but to eliminate CD4^+^ T cells with effector function before *S*. *aureus* challenge (S2 Fig). In the kidney abscess model, the depletion of CD4^+^ T cells abolished the protection conferred by 4C-Staph/T7-alum, as evidenced by the numbers of CFU found in kidneys that was not different in the T7-alum vaccine group (*p* = 0.145, Fig 5A). In contrast, CD4^+^ T cell depletion did not impact the efficacy of 4C-Staph/alum (statistically significant decrease in CFU in kidneys vs. mice vaccinated with alum, *p* = 0.002). In the peritonitis model, depletion of CD4^+^ T cells significantly decreased the survival of mice vaccinated with 4C-Staph/T7-alum (58% survival vs. 79% of mice treated with isotype control antibody, *p* = 0.034), but not of mice vaccinated with 4C-Staph/alum (13% survival vs. 31% of mice treated with isotype control antibody, *p* = 0.207, Fig 5B). In addition, depletion of CD4^+^ T cells in mice vaccinated with 4C-Staph/T7-alum caused an increase of 1.74 log_10_ in the CFU/kidneys of survivors as compared to isotype control antibody-treated mice (*p* = 0.0372, Fig 5C).

Overall, these data show that effector CD4^+^ T cells contribute to the protection conferred by one dose of 4C-Staph vaccine adjuvanted with T7-alum both in the kidney abscess and peritonitis models, although other mechanisms of protection were involved, in agreement with the role of humoral immunity that we have shown in Fig 3.

### Neutralization of IL-17A increases the bacterial load in kidneys of mice vaccinated with 4C-Staph/T7-alum upon i.p. *S*. *aureus* infection

**Fig 6:**
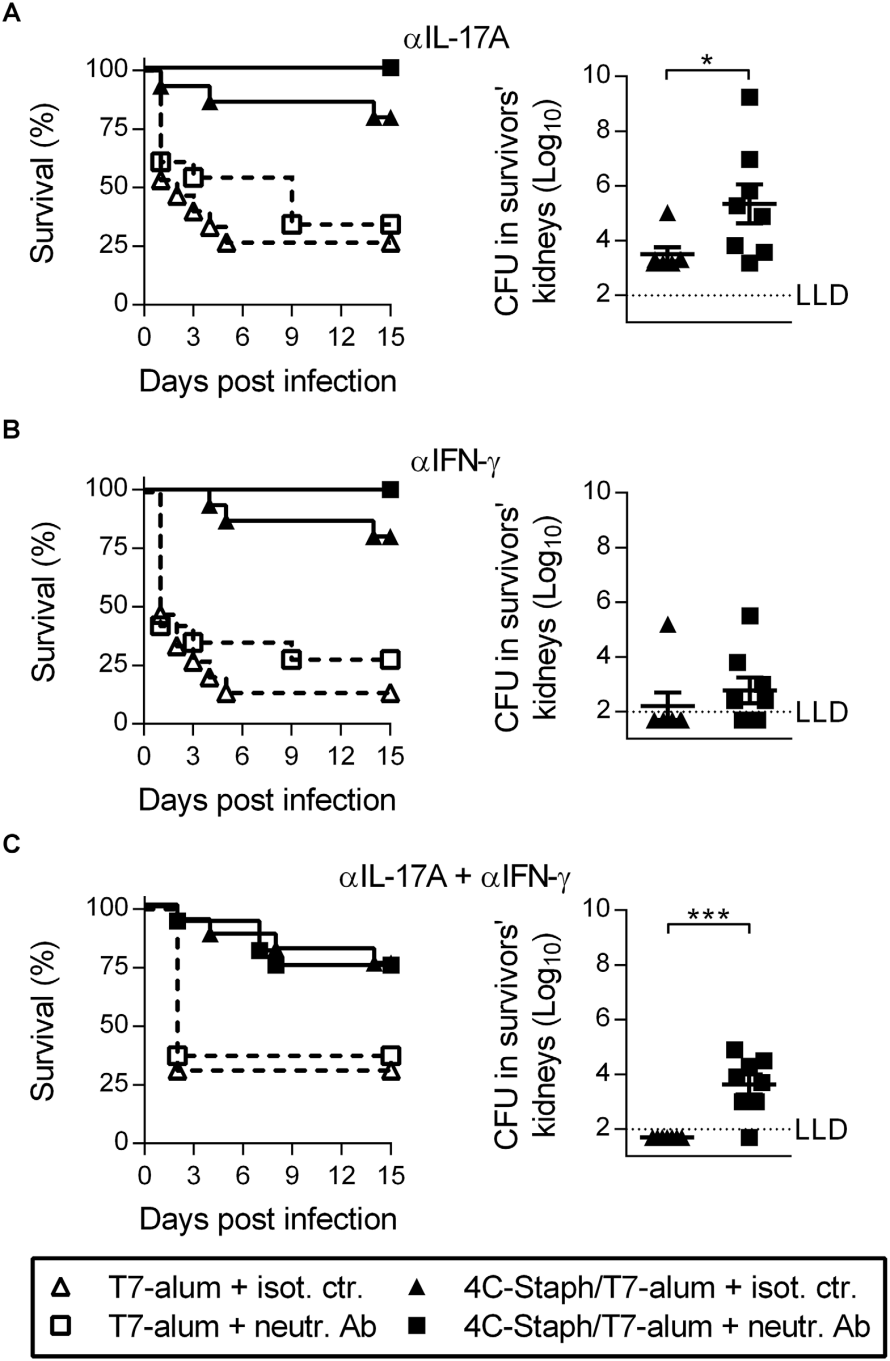
IL-17A neutralization in mice vaccinated with 4C-Staph/T7-alum has no effect on survival but increases the bacterial load in kidneys upon i.p. *S*. *aureus* challenge. Twelve days after vaccination with 4C-Staph/T7-alum or T7-alum, BALB/c mice (n = 15–16) were challenged i.p. with *S*. *aureus*. Mice were injected i.p. with neutralizing mAb (neutr. Ab): (**A**) anti-IL-17A; (**B**) anti-IFN-γ; or (**C**) anti-IL-17A and anti-IFN-γ 3 h before challenge and every other day for 15 days after challenge. Control mice were injected with isot. ctr. (**A-C**, left panels) Survival was monitored for 15 days after challenge. Data are the merge of two independent experiments. No statistically significant differences between mice vaccinated with 4C-Staph/T7-alum treated with neutr. Ab or isot. ctr. (Log-rank test). (**A-C**, right panels) Fifteen days after *S*. *aureus* inoculation, survivors (n = 7–8) were euthanized, both kidneys homogenized and CFU enumerated. Each symbol represents a mouse. Mean ± SEM. One representative experiment out of two is shown. The dotted line indicates the lower limit of CFU detection. *p < 0.01; ***p < 0.001 by unpaired Student *t* test, one-tailed.

Depletion of effector CD4^+^ T cells had no effect on protection conferred by vaccination with 4C-Staph/alum in either the kidney abscess or the peritonitis models (Fig 5A and 5B) indicating that the quality of effector CD4^+^ T cells induced by 4C-Staph/T7-alum vaccination is important. Since 4C-Staph/T7-alum induced a vaccine-specific CD4^+^ T cell response more polarized towards Th1 and Th17 cells as compared to 4C-Staph/alum (Fig 4B), we neutralized IL-17A and IFN-γ, separately or together, immediately before and repeatedly after i.p. challenge to block the effects of these cytokines produced in response to *S*. *aureus*. Treatment with neutralizing mAb specific for IL-17A and/or IFN-γ had no effect on survival compared to treatment with isotype-matched control antibody (Fig 6A–6C, left panels). However, neutralization of IL-17A alone or in combination with IFN-γ caused a 1.85 log_10_ (*p* = 0.019) and a 1.66 log_10_ (*p* = 0.0002) CFU increase, respectively, in kidneys of mice that survived the infection compared to survivors injected with isotype-matched control antibody (Fig 6A and 6C, right panels). Neutralization of IFN-γ alone did not cause a significant increase in CFU/kidneys (*p* = 0.253, Fig 6B, right panel). These data indicated that IL-17A produced in mice vaccinated with 4C-Staph/T7-alum in response to *S*. *aureus* i.p. infection is important to control dissemination of staphylococci to organs distant from the infection site.

## Discussion

A vaccine that elicits a fast protective response against *S*. *aureus* infection would be highly desirable for patients with planned surgery or in intensive care units [1–3]. Appropriately adjuvanted 4C-Staph vaccine might achieve this goal. Therefore, we formulated 4C-Staph with a novel small molecule targeting TLR7 adsorbed to alum [24] to give 4C-Staph/T7-alum, and tested this vaccine formulation as single dose in *S*. *aureus* kidney abscess and peritonitis mouse models.

We showed that 4C-Staph/T7-alum vaccine reduced by more than 100-fold the bacterial burden in kidneys of mice infected i.v. and protected roughly 80% of vaccinated mice from the lethal outcome associated with an i.p. challenge, outperforming 4C-Staph/alum formulation. Remarkably, 91% of 4C-Staph/T7-alum vaccinated mice that survived the i.p. infection had no detectable staphylococci in kidneys as compared to 54% of 4C-Staph/alum vaccinated mice. These observations on one hand showed that 4C-Staph/T7-alum promoted a more efficient control of bacteria than 4C-Staph/alum, and on the other hand highlighted that considering just survival rates as an index of *S*. *aureus* vaccine efficacy can lead to overestimated results. Indeed, the dichotomy between survival and bacterial burden has been recently highlighted by an elegant study by Maurer *et al*. [28].

The immunologic correlates of protection induced by 4C-Staph/T7-alum vaccine consisted in faster: (*i*) seroconversion of nearly 100% of vaccinated mice; (*ii*) production of antibodies specific for each of the vaccine antigens; (*iii*) induction of Hla-neutralizing antibodies; (*iv*) induction of vaccine-specific CD4^+^ T cells, a higher percentage of which produced IFN-γ, IL-17A, and TNF than those induced by 4C-Staph/alum.

Protection conferred by 4C-Staph/T7-alum was mediated by antibodies, as demonstrated by the lack of efficacy of the vaccine in B cell-deficient mice and the passive protection against *S*. *aureus* challenge achieved by transferring immune sera. Furthermore, depletion of effector CD4^+^ T cells in mice vaccinated with 4C-Staph/T7-alum, but not 4C-Staph/alum, resulted in an increase in renal bacterial burden upon i.v. or i.p. challenge as well as in reduction of survival rate upon i.p. challenge, indicating that effector CD4^+^ T cells’ polarization towards Th1 and Th17 induced by 4C-Staph/T7-alum (but not 4C-Staph/alum) vaccination is likely to be crucial. Indeed, neutralization of IL-17A, alone or together with IFN-γ (but not IFN-γ alone) resulted in a 100-fold increase in bacterial load in kidneys of 4C-Staph/T7-alum-vaccinated mice. While not statistically significant, there was a trend for an increase in CFU in IFN-γ-neutralizing antibody-treated mice and a more marked increase in CFU in mice treated with the combination of IL-17A- and IFN-γ-neutralizing antibodies compared to IL-17A-treated mice, suggesting that IFN-γ might also play a role in protection. Neutralization of either cytokine, alone or in combination, had no effect on survival. This result together with the partial effect on survival of effector CD4^+^ T cell depletion as opposed to the dramatic effect of the lack of B cells/antibodies in J_H_ mice indicated that antibodies are required at the moment of intraperitoneal infection to control bacterial growth and toxicity, giving time to effector CD4^+^ T cells to expand and exert their protective role.

The importance of the humoral response in protection against *S*. *aureus* infection is shown by preclinical as well as clinical data (reviewed in [26]). Passive transfer of antibodies raised against different staphylococcal antigens conferred partial protection against *S*. *aureus* in mouse models [8, 14, 29]. In humans, circulating antibodies to several *S*. *aureus* antigens are commonly found, particularly in colonized subjects, which present milder disease outcomes to systemic infections as compared to non-colonized patients (reviewed in [26]). In this study, passive vaccination protected all animals from death in the first days after i.p. challenge, while survival rates decreased afterwards, suggesting that a continuous supply of functional antibodies is needed, and/or mechanisms other than antibodies contribute to 4C-Staph/T7-alum-induced protection. The concept that antibodies alone are insufficient to protect against *S*. *aureus* infection is supported by the lack of efficacy of several passive and active immunization strategies in phase 3 clinical trials, despite high antibody titers were achieved [26, 30].

The role of cell-mediated immunity and in particular of IL-17-producing cells, belonging either to the innate immunity (e.g. γδ T cells) or to the adaptive immunity (i.e. Th17), in protection against *S*. *aureu*s is well documented both in patients with genetic defects that affect the IL-23/IL-17 immune axis and mice deficient in *il-17a*, *il-17f* or *il-17ra* [26, 31–33]. IL-17A and IL-17F act on many non-immune cells, including epithelial cells, inducing: (*i*) production of chemokines that in turn mobilize and recruit neutrophils and macrophages to the site of infection; and (*ii*) production of anti-microbial peptides [34]. Both mechanisms promote *S*. *aureus* clearance (reviewed in [27]). In addition, human Th17 can kill bacteria, including *S*. *aureus*, through the anti-microbial action of IL-26 that they produce [35]. In mice, IL-17 produced by γδ T cells was protective against *S*. *aureus* infection [36–39], while IL-17 produced by Th17 in response to vaccination with Als3p, ClfA, and IsdB contributed to protection against *S*. *aureus* [40–42], supporting our own observations with 4C-Staph/T7-alum.

In the peritonitis model, IL-17A neutralization and CD4^+^ effector T cell-depletion equally increased the bacterial load in kidneys of mice vaccinated with 4C-Staph/T7-alum, suggesting that Th17 are the main source of IL-17A in these settings. The contribution of IL-17-producing cells other than Th17 deserves further investigation. The fact that effector CD4^+^ T cell depletion, but not IL-17A (or IFN-γ) neutralization, affected also mice survival indicated that other cytokines produced by CD4^+^ effector T cells might be important. IL-17F, which was produced by vaccine-specific CD4^+^ T cells (data not shown) and was not neutralized by the anti-IL-17A antibody, could be such a cytokine. IL-17F contributes to control *S*. *aureus* infection since double *il-17a*^-/-^*il-17f*^-/-^ deficient mice, but not single *il-17a*^-/-^ or *il-17f*^-/-^ deficient mice, were more susceptible to opportunistic *S*. *aureus* infections [43]. TNF could also be important since it can not only synergize with IL-17A and IL-17F, which on their own are poor activators of signaling [44, 45], but also prime neutrophils, rendering them faster and more efficient against pathogens [46, 47].

In summary, we showed that one dose of 4C-Staph/T7-alum vaccine elicits a fast and efficacious protection against *S*. *aureus* systemic as well as peripheral infection through the induction of vaccine-specific functional antibodies, CD4^+^ effector T cells, and IL-17A. Cooperation between humoral and cell-mediated immunity is likely to be required to achieve efficacious protection against the broad spectrum of pathologies induced by *S*. *aureus* infections.

## Supporting Information

S1 Fig Gating-tree for phenotypic and functional characterization of vaccine-specific CD4^+^ T cells by polychromatic intracellular flow cytometry. Splenocytes from single mice immunized by 12 days were stimulated or not with vaccine antigens (10 μg/ml each) *in vitro*. Splenocytes were then stained and analyzed by intracellular cytokine staining. Live cells were identified based on Live/Dead staining. Lymphocytes were gated based on their forward side scatter (FSC) vs. side scatter (SSC) profile. Singlets were gated based on their SSC properties. CD4^+^CD44^high^ T cells were identified based on CD3, CD4, and CD44 expression. Inside the CD4^+^CD44^high^ T-cell population, cells producing IL-2, TNF, IL-4/IL-13, IFN-γ or IL-17A were identified setting gates on non-stimulated cells (not shown). The dot plots refer to splenocytes of a representative mouse immunized with 4C-Staph/T7-alum stimulated *in vitro* with vaccine proteins.
(TIF)

S2 Fig Depletion efficacy of CD4^+^ T cells in peripheral blood of mice treated with anti-CD4 or isot. ctr. demonstrated by flow cytometry analyses. Blood was collected from individual mice 9 days after vaccination and CD4^+^ T cells were identified in live white blood cells based on the expression of CD3, CD4 and CD8 markers. Representative dot plots are shown.
(TIF)

## References

[1] Chen LF, Arduino JM, Sheng S, Muhlbaier LH, Kanafani ZA, Harris AD, et al Epidemiology and outcome of major postoperative infections following cardiac surgery: Risk factors and impact of pathogen type. Am J Infect Control. 2012;40(10):963–8. 10.1016/j.ajic.2012.01.012 22609237 PMC3535474

[2] Kanafani ZA, Arduino JM, Muhlbaier LH, Kaye KS, Allen KB, Carmeli Y, et al Incidence of and preoperative risk factors for *Staphylococcus aureus* bacteremia and chest wound infection after cardiac surgery. Infect Control Hosp Epidemiol. 2009;30(3):242–8. Epub 2009/02/10. 10.1086/596015 . 19199534

[3] Vincent J, Rello J, Marshall J, Silva E, Anzueto A, Martin CD, et al International study of the prevalence and outcomes of infection in intensive care units. JAMA. 2009;302(21):2323–9. 10.1001/jama.2009.1754 19952319

[4] van Hal SJ, Jensen SO, Vaska VL, Espedido BA, Paterson DL, Gosbell IB. Predictors of mortality in *Staphylococcus aureus* Bacteremia. Clin Microbiol Rev. 2012;25(2):362–86. Epub 2012/04/12. 10.1128/cmr.05022-11 ; PubMed Central PMCID: PMCPmc3346297. 22491776 PMC3346297

[5] Bagnoli F, Fontana MR, Soldaini E, Mishra RPN, Fiaschi L, Cartocci E, et al Vaccine composition formulated with a novel TLR7-dependent adjuvant induces high and broad protection against *Staphylococcus aureus*. Proc Natl Acad Sci U S A. 2015 10.1073/pnas.1424924112 PMC4378396 25775551

[6] Menzies BE, Kernodle DS. Site-directed mutagenesis of the alpha-toxin gene of *Staphylococcus aureus*: role of histidines in toxin activity in vitro and in a murine model. Infect Immun. 1994;62(5):1843–7. Epub 1994/05/01. ; PubMed Central PMCID: PMCPmc186423. 8168947 10.1128/iai.62.5.1843-1847.1994 PMC186423

[7] Berube BJ, Bubeck Wardenburg J. *Staphylococcus aureus* alpha-toxin: nearly a century of intrigue. Toxins. 2013;5(6):1140–66. Epub 2013/07/31. ; PubMed Central PMCID: PMCPmc3717774. 23888516 10.3390/toxins5061140 PMC3717774

[8] Bubeck Wardenburg J, Schneewind O. Vaccine protection against *Staphylococcus aureus* pneumonia. J Exp Med. 2008;205(2):287–94. Epub 2008/02/13. 10.1084/jem.20072208 ; PubMed Central PMCID: PMCPmc2271014. 18268041 PMC2271014

[9] Rauch S, DeDent AC, Kim HK, Bubeck Wardenburg J, Missiakas DM, Schneewind O. Abscess Formation and Alpha-Hemolysin Induced Toxicity in a Mouse Model of *Staphylococcus aureus* Peritoneal Infection. Infect Immun. 2012;80(10):3721–32. 10.1128/iai.00442-12 22802349 PMC3457571

[10] Kennedy AD, Bubeck Wardenburg J, Gardner DJ, Long D, Whitney AR, Braughton KR, et al Targeting of alpha-hemolysin by active or passive immunization decreases severity of USA300 skin infection in a mouse model. J Infect Dis. 2010;202(7):1050–8. Epub 2010/08/24. 10.1086/656043 ; PubMed Central PMCID: PMCPmc2945289. 20726702 PMC2945289

[11] Burts ML, Williams WA, DeBord K, Missiakas DM. EsxA and EsxB are secreted by an ESAT-6-like system that is required for the pathogenesis of *Staphylococcus aureus* infections. Proc Natl Acad Sci U S A. 2005;102(4):1169–74. 10.1073/pnas.0405620102 15657139 PMC545836

[12] Korea CG, Balsamo G, Pezzicoli A, Merakou C, Tavarini S, Bagnoli F, et al Staphylococcal Esx proteins modulate apoptosis and release of intracellular *Staphylococcus aureus* during infection in epithelial cells. Infect Immun. 2014;82(10):4144–53. Epub 2014/07/23. 10.1128/iai.01576-14 ; PubMed Central PMCID: PMCPmc4187876. 25047846 PMC4187876

[13] Zhang BZ, Hua YH, Yu B, Lau CC, Cai JP, Zheng SY, et al Recombinant ESAT-6-like proteins provoke protective immune responses against invasive *Staphylococcus aureus* disease in a murine model. Infect Immun. 2015;83(1):339–45. Epub 2014/11/05. 10.1128/iai.02498-14 ; PubMed Central PMCID: PMCPmc4288882. 25368117 PMC4288882

[14] Mishra RPN, Mariotti P, Fiaschi L, Nosari S, Maccari S, Liberatori S, et al *Staphylococcus aureus* FhuD2 is involved in the early phase of staphylococcal dissemination and generates protective immunity in mice. J Infect Dis. 2012;206(7):1041–9. 10.1093/infdis/jis463 22829645

[15] Mariotti P, Malito E, Biancucci M, Lo Surdo P, Mishra RP, Nardi-Dei V, et al Structural and functional characterization of the *Staphylococcus aureus* virulence factor and vaccine candidate FhuD2. Biochem J. 2013;449(3):683–93. Epub 2012/11/02. 10.1042/bj20121426 . 23113737

[16] Sebulsky MT, Heinrichs DE. Identification and characterization of fhuD1 and fhuD2, two genes involved in iron-hydroxamate uptake in *Staphylococcus aureus*. J Bacteriol. 2001;183(17):4994–5000. Epub 2001/08/08. 11489851 10.1128/JB.183.17.4994-5000.2001 PMC95374

[17] Schluepen C, Malito E, Marongiu A, Schirle M, McWhinnie E, Lo Surdo P, et al Mining the bacterial unknown proteome: identification and characterization of a novel family of highly conserved protective antigens in *Staphylococcus aureus*. Biochem J. 2013;455(3):273–84. Epub 2013/07/31. 10.1042/bj20130540 . 23895222

[18] Reed SG, Orr MT, Fox CB. Key roles of adjuvants in modern vaccines. Nat Med. 2013;19(12):1597–608. Epub 2013/12/07. 10.1038/nm.3409 . 24309663

[19] Maisonneuve C, Bertholet S, Philpott DJ, De Gregorio E. Unleashing the potential of NOD- and Toll-like agonists as vaccine adjuvants. Proc Natl Acad Sci U S A. 2014;111(34):12294–9. Epub 2014/08/20. 10.1073/pnas.1400478111 ; PubMed Central PMCID: PMCPmc4151741. 25136133 PMC4151741

[20] Annunziato F, Romagnani C, Romagnani S. The 3 major types of innate and adaptive cell-mediated effector immunity. J Allergy Clin Immunol. 2015;135(3):626–35. Epub 2014/12/22. 10.1016/j.jaci.2014.11.001 . 25528359

[21] Medzhitov R, Janeway CA Jr. Innate Immunity: The Virtues of a Nonclonal System of Recognition. Cell. 1997;91(3):295–8. 10.1016/S0092-8674(00)80412-2 9363937

[22] Akira S, Takeda K. Toll-like receptor signalling. Nat Rev Immunol. 2004;4(7):499–511. Epub 2004/07/02. 10.1038/nri1391 . 15229469

[23] Vasilakos JP, Tomai MA. The use of Toll-like receptor 7/8 agonists as vaccine adjuvants. Expert Rev Vaccines. 2013;12(7):809–19. 10.1586/14760584.2013.811208 23885825

[24] Wu TY, Singh M, Miller AT, De Gregorio E, Doro F, D'Oro U, et al Rational design of small molecules as vaccine adjuvants. Sci Transl Med. 2014;6(263):263ra160 Epub 2014/11/21. 10.1126/scitranslmed.3009980 . 25411473

[25] Chen J, Trounstine M, Alt FW, Young F, Kurahara C, Loring JF, et al Immunoglobulin gene rearrangement in B cell deficient mice generated by targeted deletion of the JH locus. Int Immunol. 1993;5(6):647–56. Epub 1993/06/01. . 8347558 10.1093/intimm/5.6.647

[26] Pozzi C, Lofano G, Mancini F, Soldaini E, Speziale P, De Gregorio E, et al Phagocyte subsets and lymphocyte clonal deletion behind ineffective immune response to *Staphylococcus aureus*. FEMS Microbiol Rev. 2015 Epub 2015/05/23. 10.1093/femsre/fuv024 . 25994610

[27] Proctor RA. Challenges for a universal *Staphylococcus aureus* vaccine. Clin Infect Dis. 2012;54(8):1179–86. Epub 2012/02/23. 10.1093/cid/cis033 . 22354924

[28] Maurer K, Reyes-Robles T, Alonzo F 3rd, Durbin J, Torres VJ, Cadwell K. Autophagy mediates tolerance to *Staphylococcus aureus* alpha-toxin. Cell Host Microbe. 2015;17(4):429–40. Epub 2015/03/31. 10.1016/j.chom.2015.03.001 ; PubMed Central PMCID: PMCPmc4392646. 25816775 PMC4392646

[29] Kim HK, DeDent A, Cheng AG, McAdow M, Bagnoli F, Missiakas DM, et al IsdA and IsdB antibodies protect mice against *Staphylococcus aureus* abscess formation and lethal challenge. Vaccine. 2010;28(38):6382–92. Epub 2010/03/17. 10.1016/j.vaccine.2010.02.097 ; PubMed Central PMCID: PMCPmc3095377. 20226248 PMC3095377

[30] Fattom A, Matalon A, Buerkert J, Taylor K, Damaso S, Boutriau D. Efficacy profile of a bivalent *Staphylococcus aureus* glycoconjugated vaccine in adults on hemodialysis: Phase III randomized study. Hum Vaccin Immunother. 2015;11(3):632–41. Epub 2014/12/09. 10.4161/hv.34414 . 25483694 PMC4514248

[31] Cypowyj S, Picard C, Marodi L, Casanova JL, Puel A. Immunity to infection in IL-17-deficient mice and humans. Eur J Immunol. 2012;42(9):2246–54. Epub 2012/09/06. 10.1002/eji.201242605 ; PubMed Central PMCID: PMCPmc3720135. 22949323 PMC3720135

[32] Lanternier F, Cypowyj S, Picard C, Bustamante J, Lortholary O, Casanova JL, et al Primary immunodeficiencies underlying fungal infections. Curr Opin Pediatr. 2013;25(6):736–47. Epub 2013/11/19. 10.1097/mop.0000000000000031 ; PubMed Central PMCID: PMCPmc4098727. 24240293 PMC4098727

[33] Gaffen SL, Jain R, Garg AV, Cua DJ. The IL-23-IL-17 immune axis: from mechanisms to therapeutic testing. Nat Rev Immunol. 2014;14(9):585–600. Epub 2014/08/26. 10.1038/nri3707 ; PubMed Central PMCID: PMCPmc4281037. 25145755 PMC4281037

[34] Ye P, Rodriguez FH, Kanaly S, Stocking KL, Schurr J, Schwarzenberger P, et al Requirement of interleukin 17 receptor signaling for lung CXC chemokine and granulocyte colony-stimulating factor expression, neutrophil recruitment, and host defense. J Exp Med. 2001;194(4):519–27. Epub 2001/08/22. ; PubMed Central PMCID: PMCPmc2193502. 11514607 10.1084/jem.194.4.519 PMC2193502

[35] Meller S, Di Domizio J, Voo KS, Friedrich HC, Chamilos G, Ganguly D, et al TH17 cells promote microbial killing and innate immune sensing of DNA via interleukin 26. Nat Immunol. 2015;16(9):970–9. Epub 2015/07/15. 10.1038/ni.3211 . 26168081 PMC4776746

[36] Cho JS, Pietras EM, Garcia NC, Ramos RI, Farzam DM, Monroe HR, et al IL-17 is essential for host defense against cutaneous *Staphylococcus aureus* infection in mice. J Clin Invest. 2010;120(5):1762–73. Epub 2010/04/07. 10.1172/jci40891 20364087 PMC2860944

[37] Cheng AG, Kim HK, Burts ML, Krausz T, Schneewind O, Missiakas DM. Genetic requirements for *Staphylococcus aureus* abscess formation and persistence in host tissues. FASEB J. 2009;23(10):3393–404. 10.1096/fj.09-135467 19525403 PMC2747682

[38] Maher BM, Mulcahy ME, Murphy AG, Wilk M, O'Keeffe KM, Geoghegan JA, et al Nlrp-3-driven interleukin 17 production by gammadeltaT cells controls infection outcomes during *Staphylococcus aureus* surgical site infection. Infect Immun. 2013;81(12):4478–89. Epub 2013/10/02. 10.1128/iai.01026-13 ; PubMed Central PMCID: PMCPmc3837970. 24082072 PMC3837970

[39] Murphy AG, O'Keeffe KM, Lalor SJ, Maher BM, Mills KH, McLoughlin RM. *Staphylococcus aureus* infection of mice expands a population of memory gammadelta T cells that are protective against subsequent infection. J Immunol. 2014;192(8):3697–708. Epub 2014/03/14. 10.4049/jimmunol.1303420 ; PubMed Central PMCID: PMCPmc3979672. 24623128 PMC3979672

[40] Joshi A, Pancari G, Cope L, Bowman EP, Cua D, Proctor RA, et al Immunization with Staphylococcus aureus iron regulated surface determinant B (IsdB) confers protection via Th17/IL17 pathway in a murine sepsis model. Hum Vaccin Immunother. 2012;8(3):336–46. 10.4161/hv.18946 22327491 PMC3426080

[41] Lin L, Ibrahim AS, Xu X, Farber JM, Avanesian V, Baquir B, et al Th1-Th17 cells mediate protective adaptive immunity against *Staphylococcus aureus* and *Candida albicans* infection in mice. PLoS Pathog. 2009;5(12):e1000703 10.1371/journal.ppat.1000703 20041174 PMC2792038

[42] Narita K, Hu D-L, Mori F, Wakabayashi K, Iwakura Y, Nakane A. Role of Interleukin-17A in Cell-Mediated Protection against *Staphylococcus aureus* Infection in Mice Immunized with the Fibrinogen-Binding Domain of Clumping Factor A. Infect Immun. 2010;78(10):4234–42. 10.1128/iai.00447-10 20679443 PMC2950370

[43] Ishigame H, Kakuta S, Nagai T, Kadoki M, Nambu A, Komiyama Y, et al Differential roles of interleukin-17A and -17F in host defense against mucoepithelial bacterial infection and allergic responses. Immunity. 2009;30(1):108–19. Epub 2009/01/16. 10.1016/j.immuni.2008.11.009 . 19144317

[44] Qian Y, Liu C, Hartupee J, Altuntas CZ, Gulen MF, Jane-wit D, et al The adaptor Act1 is required for interleukin 17-dependent signaling associated with autoimmune and inflammatory disease. Nat Immunol. 2007;8(3):247–56. doi: http://www.nature.com/ni/journal/v8/n3/suppinfo/ni1439_S1.html. 17277779 10.1038/ni1439

[45] Gaffen SL. Structure and signalling in the IL-17 receptor family. Nat Rev Immunol. 2009;9(8):556–67. 10.1038/nri2586 19575028 PMC2821718

[46] van Kessel KP, Bestebroer J, van Strijp JA. Neutrophil-Mediated Phagocytosis of Staphylococcus aureus. Front Immunol. 2014;5:467 Epub 2014/10/14. 10.3389/fimmu.2014.00467 ; PubMed Central PMCID: PMCPmc4176147. 25309547 PMC4176147

[47] von Köckritz-Blickwede M, Rohde M, Oehmcke S, Miller LS, Cheung AL, Herwald H, et al Immunological mechanisms underlying the genetic predisposition to severe *Staphylococcus aureus* infection in the mouse model. Am J Pathol. 2008;173(6):1657–68. 10.2353/ajpath.2008.080337 18974303 PMC2626378

