# Supplementary figures and images for: One Dose of Staphylococcus aureus 4C-Staph Vaccine Formulated with a Novel TLR7-Dependent Adjuvant Rapidly Protects Mice through Antibodies, Effector CD4+ T Cells, and IL-17A
Source: PLoS One. 2016 Jan 26;11(1):e0147767. doi: 10.1371/journal.pone.0147767 (PMC4727907; doi:10.1371/journal.pone.0147767)

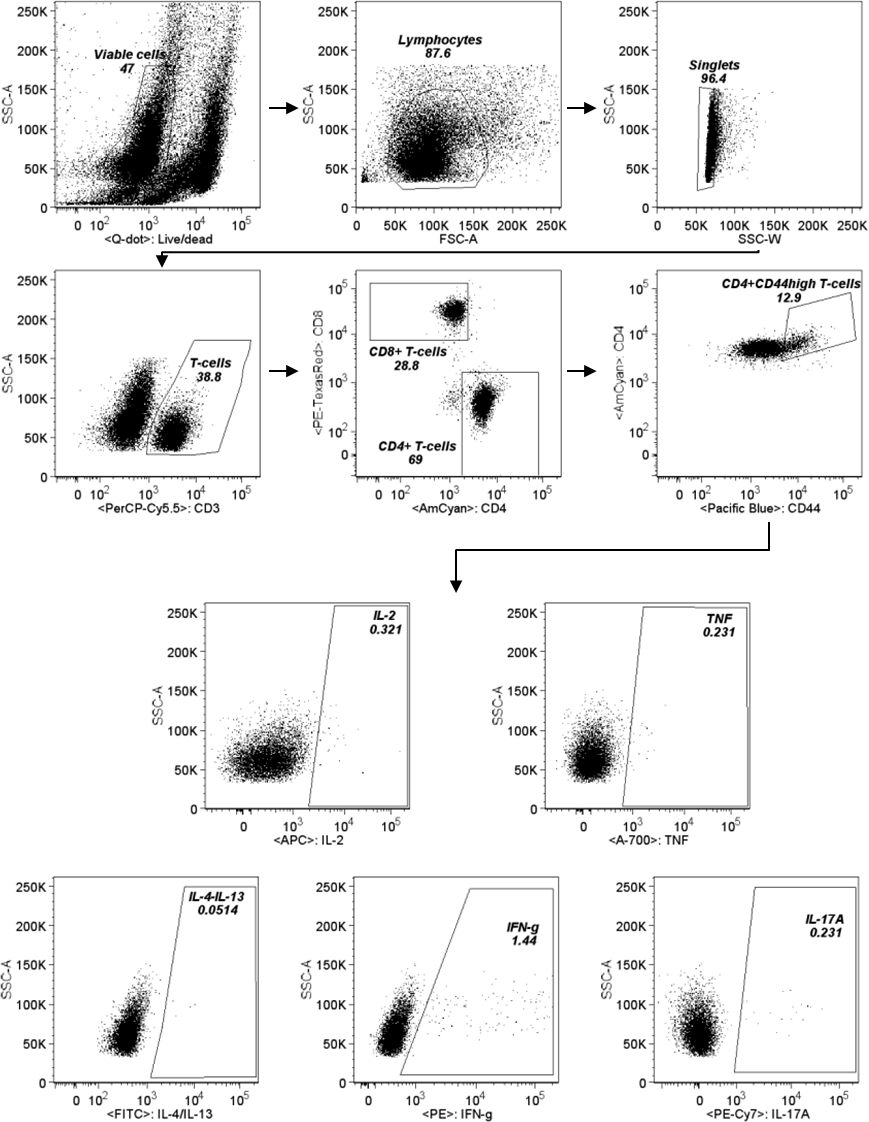

Supplement: S1 Fig — Splenocytes from single mice immunized by 12 days were stimulated or not with vaccine antigens (10 μg/ml each) in vitro. Splenocytes were then stained and analyzed by intracellular cytokine staining. Live cells were identified based on Live/Dead staining. Lymphocytes were gated based on their forward side scatter (FSC) vs. side scatter (SSC) profile. Singlets were gated based on their SSC properties. CD4+CD44high T cells were identified based on CD3, CD4, and CD44 expression. Inside the CD4+CD44high T-cell population, cells producing IL-2, TNF, IL-4/IL-13, IFN-γ or IL-17A were identified setting gates on non-stimulated cells (not shown). The dot plots refer to splenocytes of a representative mouse immunized with 4C-Staph/T7-alum stimulated in vitro with vaccine proteins. (TIF) [file pone.0147767.s001.tif]

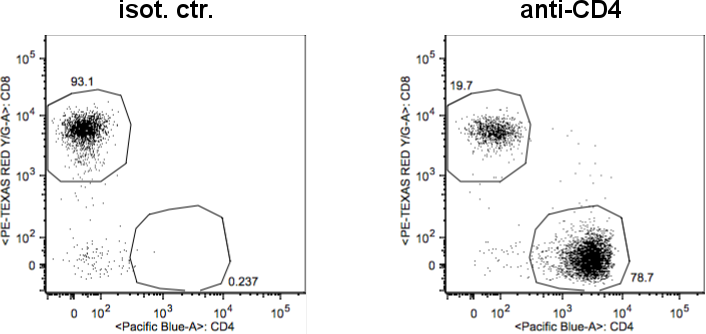

Supplement: S2 Fig — Blood was collected from individual mice 9 days after vaccination and CD4+ T cells were identified in live white blood cells based on the expression of CD3, CD4 and CD8 markers. Representative dot plots are shown. (TIF) [file pone.0147767.s002.tif]
